# Preclinical efficacy studies in investigator brochures: Do they enable risk–benefit assessment?

**DOI:** 10.1371/journal.pbio.2004879

**Published:** 2018-04-05

**Authors:** Susanne Wieschowski, William Wei Lim Chin, Carole Federico, Sören Sievers, Jonathan Kimmelman, Daniel Strech

**Affiliations:** 1 Institute for Ethics, History, and Philosophy of Medicine, Hannover Medical School, Hannover, Germany; 2 Studies of Translation, Ethics, and Medicine (STREAM), Biomedical Ethics Unit, McGill University, Montreal, Québec, Canada; University of Edinburgh, United Kingdom of Great Britain and Northern Ireland

## Abstract

Human protection policies require favorable risk–benefit judgments prior to launch of clinical trials. For phase I and II trials, evidence for such judgment often stems from preclinical efficacy studies (PCESs). We undertook a systematic investigation of application materials (investigator brochures [IBs]) presented for ethics review for phase I and II trials to assess the content and properties of PCESs contained in them. Using a sample of 109 IBs most recently approved at 3 institutional review boards based at German Medical Faculties between the years 2010–2016, we identified 708 unique PCESs. We then rated all identified PCESs for their reporting on study elements that help to address validity threats, whether they referenced published reports, and the direction of their results. Altogether, the 109 IBs reported on 708 PCESs. Less than 5% of all PCESs described elements essential for reducing validity threats such as randomization, sample size calculation, and blinded outcome assessment. For most PCESs (89%), no reference to a published report was provided. Only 6% of all PCESs reported an outcome demonstrating no effect. For the majority of IBs (82%), all PCESs were described as reporting positive findings. Our results show that most IBs for phase I/II studies did not allow evaluators to systematically appraise the strength of the supporting preclinical findings. The very rare reporting of PCESs that demonstrated no effect raises concerns about potential design or reporting biases. Poor PCES design and reporting thwart risk–benefit evaluation during ethical review of phase I/II studies.

## Author summary

To make a clinical trial ethical, regulatory agencies and institutional review boards have to judge whether the trial-related benefits (the knowledge gain) outweigh the trial-inherent risks. For early-phase human research, these risk–benefit assessments are often based on evidence from preclinical animal studies reported in so-called “investigator brochures.” However, our analysis shows that the vast majority of such investigator brochures lack sufficient information to systematically appraise the strength of the supporting preclinical findings. Furthermore, the very rare reporting of preclinical efficacy studies that demonstrated no effect raises concerns about potential design and/or reporting biases. The poor preclinical study design and reporting thwarts risk–benefit evaluation during ethical review of early human research. Regulators should develop standards for the design and reporting of preclinical efficacy studies in order to support the conduct of ethical clinical trials.

## Introduction

Early phase human studies (phase I and II trials) aim to establish the safety, rationale, and conditions for testing new drugs in rigorous, randomized controlled phase III trials. Because early phase human studies expose human research subjects to unproven—and in some cases previously untested—interventions, they present major human protection challenges [1]. Key to meeting these challenges is establishing a favorable risk–benefit ratio in prospective ethical review.

In early phase trials, assessment of risks and benefits depends heavily on evidence gathered in preclinical animal studies. Over the past 10 years, many commentators have raised concerns about the design and reporting of preclinical reports [2–9]. These concerns have been mainly informed by cross-sectional studies of peer-reviewed publications [2,10] and study protocols [11] for preclinical studies. These analyses consistently show infrequent reporting of measures aimed at reducing bias, including a priori sample size calculation, blinding of outcome assessment, and randomization. Further analyses suggest that publication bias frequently leads to inflated estimation effect sizes [2].

However, many such analyses reflect preclinical studies that have been submitted for animal care committee review or that are described in publications. Many such studies are not necessarily embedded within drug development programs and may have been pursued after a drug had already shown efficacy in trials. In contrast, little is known about the extent, quality, and accessibility of preclinical evidence used to justify and review the launch of early phase clinical trials. As a result, it is unclear whether preclinical studies submitted to institutional review boards (IRBs) or regulatory agencies are described in ways that enable the respective evaluators to perform a critical assessment about the strength of evidence supporting a new trial. Nor is it clear whether such materials adhere to various standards and guidelines on the design of preclinical studies [12–14].

Clinical investigators, IRBs, data safety and monitoring boards, and regulatory agencies (e.g., the European Medicines Agency [EMA] and the Food and Drug Administration [FDA]) are all charged with risk–benefit assessment. Their main source of information is the investigator brochure (IB). According to the ICH (International Council for Harmonisation of Technical Requirements for Pharmaceuticals for Human Use) Guideline for Good Clinical Practice E6 [15], the information in IBs “should be presented in a concise, simple, objective, balanced, and non-promotional form that enables a clinician, or potential investigator, to understand it and make his/her own unbiased risk–benefit assessment of the appropriateness of the proposed trial”.

In general, the preclinical safety studies (mainly pharmacokinetics and toxicology experiments) inform judgments about risk in early phase trials. Judgments about clinical promise rely heavily on preclinical efficacy studies (PCESs, often described as “preclinical pharmacodynamic” studies in regulatory documents), which aim at providing a readout of disease response in animal models. The primary objective of this study was to determine the extent, quality, and accessibility of PCESs that are contained within IBs submitted for ethical review of early-phase clinical trials.

## Results

### Characteristics of IBs and PCESs

Altogether, we obtained 109 IBs for phase I (*n* = 15), phase I/II (*n* = 10), and phase II (*n* = 84) clinical trials that were submitted to 1 of 3 German IRBs (see the “Materials and methods” section). The majority of these IBs (*n* = 97, 83%) reflected the full sample of IBs for phase I/II trials submitted to 1 of the 3 IRBs between 2010 and 2016. The IBs covered 8 out of 12 therapeutic areas as distinguished by the European Medicine Agency (Table 1). Seven studies (6%) were “first in human,” whereas all other IBs (94%) mentioned at least some clinical evidence for the investigational product. All trials were privately funded (1 IRB did not allow recording of the funders of the 6 IBs they shared, so we have this information for only 103 IBs). These included 48 IBs (47%) from the top 25 pharma companies by global sales [16].

**Table 1:** Characteristics of investigator brochures (IBs) grouped according to the therapeutic areas as defined by the European Medicine Agency.

Therapeutic area	PCESs		IBs	
	*n*	%	*n*	%
Blood product and biotech	74	10%	6	6%
Antineoplastic and immune-modulating agents	437	62%	55	50%
Respiratory system	59	8%	6	6%
Rheumatology	10	1%	4	4%
Dermatologicals	10	1%	2	2%
Alimentary tract and metabolism	33	5%	7	6%
Anti-infectives	48	7%	20	18%
Allergy and immunology	9	1%	3	3%
Nervous system	17	2%	3	3%
Blood and blood-forming organs	6	1%	2	2%
Cardiovascular	5	1%	1	1%
Total	708	100%	109	100%

Abbreviations: PCES, preclinical efficacy study.

**Fig 1:**
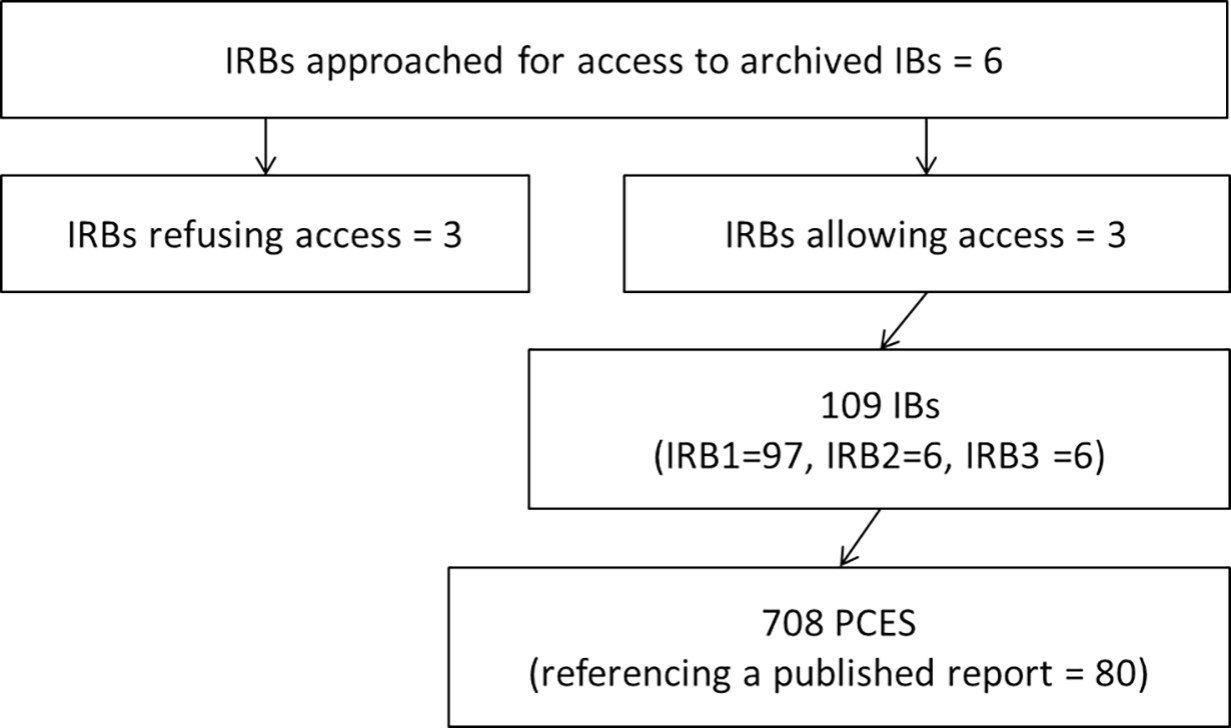
Flowchart for retrieval of preclinical efficacy studies. Abbreviations: IB, investigator brochure (for phase I/II clinical trials); IRB, institutional review board; PCES, preclinical efficacy studies (in vivo).

A total of 708 PCES were identified from all 109 IBs. The median number of PCESs per IB was 5, with 18 IBs (17%) including 0 PCESs and 10 IBs (9%) including more than 15 PCESs (max = 32 PCESs). See Fig 1.

### Reporting on practices used to address validity threats

Table 2 presents the extent to which the 109 IBs described the implementation of practices to reduce validity threats for the 708 PCESs contained in them. A sample size was reported in 26% of PCESs (*n* = 184), with a median group size of 8 animals. Sample size calculation was never explained. None of the 708 PCESs were described as using blinded treatment allocation and/or outcome assessment. Only 4% of PCESs (*n* = 26) were described as using randomization, and 5% (*n* = 38 studies) reported the exclusion of animal data.

**Table 2:** Reporting on internal, construct, and external validity items on the preclinical efficacy study (PCES) level and the investigator brochure (IB) level.

Validity items	Descriptors	All IRBs		First in human studies		IRB 1		IRB 2		IRB 3		IRB 1: Subset with reference check		IRB 1: Results of reference check	
		*N*	%	*N*	%	*N*	%	*N*	%	*N*	%	*N*	%	*N*	%
Validity items: PCES level		Total = 708 PCESs		Total = 30 PCESs		Total = 668 PCESs		Total = 15 PCESs		Total = 25 PCESs		Total = 80 PCESs		Total = 80 PCESs	
Sample size^1^	Is sample size reported?	184	26	12	40	164	25	15	100	5	20	8	10	73	91
	If yes, is sample size calculation reported?	0	0	0	0	0	0	0	0	0	0	0	0	0	0
Randomisation^1^	Is randomization reported?	26	4	4	13	22	3	0	0	4	16	1	1	22	28
Baseline characterization	Is baseline characterization reported?	127	18	19	63	109	16	4	27	14	56	5	6	61	76
Blinded assessment ^1^	Is blinding for treatment allocation reported?	0	0	0	0	0	0	0	0	0	0	0	0	0	0
	Is blinding for outcome assessment reported?	0	0	0	0	0	0	0	0	0	0	0	0	4	5
Exclusion of data from analysis^1^	Is exclusion of data reported?	38	5	1	3	38	6	0	0	0	0	4	5	11	14
Controls^1^	Is the control group reported?	329	46	24	80	290	43	14	93	25	100	30	38	78	98
Model choice^2^	Is the species of animal reported?	622	88	30	100	583	87	15	100	24	96	69	86	80	100
	Is the type of model reported?	684	97	29	97	644	96	15	100	25	100	73	91	80	100
	Is the specification of the model reported?	193	27	15	50	166	25	8	53	19	76	12	15	74	93
Outcome choice^2^	Is the outcome choice reported?	575	81	24	80	537	80	15	100	23	92	67	84	80	100
Validity items: IB level		Total = 91 IBs		Total = 7 IBs		Total = 79 IBs		Total = 6 IBs		Total = 6 IBs		Total = 20 IBs		Total = 20 IBs	
Dose response^1^	Is dose response reported?	67	74	3	43	58	73	4	67	5	83	12	60	13	65
	If yes, is positive response reported?	67	100	3	100	58	100	4	100	5	100	12	100	13	100
Age matched to patients^2^	Is the age of animals reported?	14	15	2	29	13	16	1	17	0	0	3	15	14	70
	Is the age of animals matched to patients?	8	57	1	50	8	62	0	0	0	0	1	33	1	7
Mechanistic evidence^2^	Is there evidence of the drug mechanism?	64	70	6	86	54	68	5	83	5	83	15	75	15	75
Replication of experiment^3^	Is there replication of the experiment?	27	30	4	57	24	30	1	17	2	33	8	40	8	40
Replication in different model^3^	Is there replication in a different model?	70	77	4	57	61	77	4	67	5	83	18	90	19	95
Replication in different species^3^	Is there replication in a different species?	25	27	3	43	22	28	1	17	2	33	8	40	8	40

Abbreviations: IRB, institutional review board.

^1^Internal validity item

^2^Construct validity item

^3^External validity item.

Baseline characterization of animals was described for 18% of all PCESs (*n* = 127 studies). The animal species was reported for 88% of all PCESs (*n* = 622 studies); see Table 3 for further details on animal species. The animal model used in the experiment was reported in general terms (e.g., “xenograft” or “T-cell tolerance model”) for 97% of all PCESs (*n* = 684 studies), but its specifications (e.g., transplanted cell line for xenograft or tumor size before treatment) were reported for only 27% of all PCESs (*n* = 193 studies). Outcome choice was reported for 81% of all PCESs (*n* = 575 studies).

**Table 3:** Species of included preclinical efficacy studies (PCESs).

Species	Number of PCESs	% of PCESs
Mouse/rat	579	81.8
Rabbit/hamster/guinea pig/ferret	24	3.4
Dog/pig/chicken	10	1.4
Monkey/primate	9	1.3
Not reported	86	12.1
Total	708	100.0

As described in the “Materials and methods” section, some validity issues pertain less to individual PCESs than to a package of several PCESs within 1 IB. These were thus rated at the IB level only. The following percentages refer to the 91 IBs that included at least 1 PCES. At the IB level, 9% (*n* = 8) reported for at least 1 PCES whether the age of animals matched the patient group proposed in the clinical trial. Most IBs (74%, *n* = 67) described a preclinical dose response for at least 1 studied outcome. At least 1 PCES described mechanistic evidence of efficacy in 70% of all IBs (*n* = 64). At least 1 replication of an efficacy experiment was described in 82% of IBs (*n* = 75). These included 70 replications in different models (77%) and 25 replications in a different species (27%).

### References to published preclinical evidence

A reference to published, peer-reviewed reports of preclinical efficacy was provided for 80 PCESs (11% of all PCESs) stemming from 20 IBs (18% of all IBs). These journal publications provided additional information on sample size (for 91% of PCESs reported in journals versus for 26% of PCESs reported in IBs), baseline characterization (76% versus 18%), control groups (98% versus 46%), randomization (28% versus 4%), and blinding of outcome assessment (5% versus 0%). However, no or even less additional information in journal publications was found, for example, for sample size calculations (0% for PCES in journals and 0% for PCES in IBs) and for age matching of animals and patient group (5% versus 9%). For further information, see Table 2.

### Effects reported in PCESs

Less than half of all PCESs (44%) stemming from 68 IBs (75%) reported results in quantitative terms that allowed scoring for the results as “demonstrating an effect” or “demonstrating no effect.” Altogether, 30% of all PCESs (*n* = 211) reported an effect size, and 23% of all PCES (*n* = 161) reported a *p*-value. Another 53% of all PCES (*n* = 372) provided narrative descriptions of results.

With regard to outcomes, the results for 636 PCESs (90%) demonstrated an effect, and the results for 43 PCESs (6%) demonstrated no effect. For 29 PCESs (4%), the direction of results was unclear. The 43 PCESs demonstrating no effect came from 16 IBs (18%). S1 Table gives examples for PCESs demonstrating no effect.

## Discussion

Our analysis of 109 IBs for phase I/II trials uncovered 3 striking features of the 708 PCESs the IBs presented to IRBs and regulatory agencies to support early-phase trials. First, 89% of all PCESs present data without a reference to a published, peer-reviewed report. While it is possible that sponsors maintain internal review mechanisms for PCESs, members of IRBs or regulatory agencies reviewing IBs have no way of knowing whether preclinical efficacy data have been subject to critical and independent evaluation. Further, IRBs and regulatory sponsors have no way of directly accessing preclinical reports if they are not published. Such commonplace nonpublication of preclinical evidence is potentially inconsistent with numerous scientific and ethical guidelines on early-phase trial launch [13,17].

The second finding is that much of the information needed for a favorable appraisal of the PCES’s validity is not provided in IBs. On the positive side of the ledger, IBs often contain PCESs that characterize the mechanism of action for new drugs or a dose response. Many also contain more than 1 study testing similar hypotheses, thus establishing some level of reproducibility for efficacy claims. On the negative side, IBs contain very little information that would enable reviewers to evaluate the risk of bias in these individual studies. For example, less than 20% of PCESs reported baseline characterization, exclusion of data from analysis, or randomization. Sample size calculation and blinding for outcome assessment were never reported. Many have previously argued that internal validity is the sina qua non of a valid experimental claim [18]. The dose response, mechanism, and replication studies described in IBs are difficult to interpret without knowing how well they implemented measures to limit bias and the effects of random variation. A potential reporting bias for studies demonstrating the intended mechanism or dose response would further aggravate this difficulty.

This leads us to the third striking finding: the scarcity of PCESs in IBs that do not demonstrate an effect (*n* = 43, 6%). Several nonexclusive explanations for this imbalance of outcomes in PCESs can be envisioned. One is biased study design. It is possible that in the absence of prespecifying end points or the limited use of techniques like blinded outcome assessment, PCESs consistently show large effects. A second explanation is biased inclusion of PCESs in IBs. With a median group size of 8, the PCESs in our sample had a limited ability to measure treatment effects precisely. This might have resulted in studies that showed unusually large effects. Attrition of animals in small experiments might aggravate the tendency for studies to occasionally produce large effects [19]. A recent investigation from the British Medical Journal (BMJ) supports the notion that animal data are sometimes reported selectively in IBs [20]. A third explanation is that only those treatments that show consistently positive effects in PCES were selected for early-phase trials. Though our study does not allow us to discriminate between these explanations, we think the latter explanation is improbable. Studies demonstrating no effect are crucial for demarcating the boundaries of dosing, diagnostic eligibility, or treatment timing for a new treatment [21]. The evidentiary basis for such boundaries would be important to include in an IB. Indeed, some PCESs we found (see S1 Table) demonstrated such efforts at “demarcation.”

Our study has several limitations. First, because of difficulties accessing IBs [20,22], our analysis did not utilize a random sample. Nevertheless, we believe our findings are likely to be generalizable to other IBs used for early-phase trials. For example, IBs used in our study covered a broad spectrum of different funders and addressed many different therapeutic areas. It is also important to note that the same IBs that funders submit to local IRBs are also submitted to the national regulatory agency (Bundesinstitut fuer Arzneimittel und Medizinprodukte [BfArM]). If the studies in our sample deviate from norms that are used elsewhere, these deviations fall within the window of acceptability for drug regulators.

A second limitation might be seen in the fact that only a small minority of included IBs were “first in human” studies (7 IBs, comprising 30 PCESs). However, many phase I trials that are not first in human involve new disease indications or drug combinations; PCESs are critical for justifying such studies. Moreover, phase II trials represent the first attempt to test a drug’s efficacy in human beings. Their justification ultimately rests on the evidence of clinical promise established in PCESs. In general, if information on preclinical efficacy is considered important to include in an IB, then validity reporting for the respective PCESs should be important as well.

Last, there are limitations to the way we measured the degree to which validity threats were described as being addressed in IBs. For instance, our 14-item matrix did not weight any practices based on their potential impact on bias. Also, that measures aimed at strengthening the validity of PCES findings are reported so infrequently in IBs does not necessarily mean that such measures were not implemented. Nevertheless, our matrix was based on systematic review evidence and thus provided a reasonable starting point for describing how study designs were reported in IBs. Stakeholders tasked with risk–benefit assessment, such as investigators, IRBs, regulatory agencies, and data safety and monitoring boards, have no way of knowing how studies were performed if the relevant study design information is not provided in IBs—this is especially the case if the studies themselves have not been published.

To improve the effective use of preclinical information for risk–benefit assessment in phase I/II trials, we offer the following recommendations. First, IBs should describe measures taken in PCESs to support clinical generalizability. Animal Research: Reporting of In Vivo Experiments (ARRIVE) recommendations, established in 2010, provide one suggestion for how to do so [23]. To facilitate efficient evaluation of IBs, it might be helpful to present relevant practices, like use of randomization or choice of endpoints, in tabular form. Furthermore, explicit remarks on the “level of evidence” for each preclinical study might be presented in such tables, with studies designated as “confirmatory” when they had prespecified hypotheses and protocols or “exploratory” when their hypotheses and protocols were not established prospectively. “Confirmatory” studies should also undertake the expense of employing methods that would enhance internal and construct validity−namely, a priori sample size calculation, concealed allocation, or blinded outcome assessment and use of clinically relevant endpoints [12]. Information on the reproducibility of confirmatory preclinical studies and meta-analysis of sufficiently similar studies might further improve the level of evidence [24]. A more stepwise presentation of preclinical evidence could further help evaluators to navigate through the most important questions to assess clinical promise and safety [9,25]: Have effects been reproduced in different models and/or in independent laboratories? Do the conditions of the experiment (for instance, age of animal models, timing of treatments, and outcomes) match clinical scenarios?

Second, IBs should state whether they are presenting the totality of preclinical evidence, and if not, how data were selected for inclusion in the IB. One option would be to only present preclinical studies that have been preregistered [26,27]. This increased transparency might help with preventing selective outcome reporting, and it allows evaluators to check whether other relevant preclinical studies exist.

Future studies need to evaluate how improved reporting for preclinical data presented in IBs influences risk–benefit analysis during ethical review. However, better reporting alone is unlikely to solve problems related to risk of bias in preclinical evidence. Regulatory bodies like the FDA and the EMA offer specific recommendations for the design of preclinical safety studies [28]. To our knowledge, there are no regulatory guidelines offering standards for the design and reporting of PCESs. As the IBs investigated in this study inform ethical as well as regulatory review, we recommend that regulators develop standards for the design and reporting of PCESs to be included in IBs.

## Materials and methods

### Sampling of IBs

IBs and study protocols submitted to IRBs in support of trials are difficult to access, because academic medical centers maintain IBs in strict confidence. Indeed, the difficulty of protocol access has been addressed as a major challenge for metaresearch and quality assurance of ethics review [22]. A recent BMJ investigation illustrated the challenges of accessing study protocols and IBs [20]. Because our inquiry was sensitive and protocol access is so restricted, we deemed it unlikely that randomly identifying centers for inquiry would be productive and produce a sample that was anything approaching random. We therefore approached 6 chairs of German IRBs personally to outline the rationale for our interest in analyzing the reporting of preclinical evidence in IBs. Three chairs were willing to grant access under the data protection conditions described below.

One IRB responsible for reviewing all clinical trials to be conducted at one of the leading German university hospitals gave us access to their full sample of all 97 phase I/II trials that they approved between 2010 and 2016. The IRBs at 2 other German university hospitals allowed us to access the 6most recently reviewed IBs for phase I/II trials.

### Data protection

All IBs were analyzed on-site at the 3 universities. All members of the research team signed confidentiality agreements. Results are reported in an aggregated manner and do not allow the identification of investigational products, sponsors, investigators, or other commercially sensitive information.

### Selection and rating of PCESs

To select all PCESs from an IB for coding, we applied the following inclusion criteria: (A) studies were conducted in nonhuman animals and (B) relevant to interpreting the efficacy of the investigational product (e.g., molecular, behavioral, or physiological readouts that were described as correlating with clinical activity). We excluded preclinical studies if they were (A) pharmacokinetic studies only, (B) safety and toxicology studies only, or (C) in vitro/ex vivo studies.

To rate the degree to which the included PCESs addressed threats to valid clinical inference, a matrix was employed based on results from a systematic review of 26 guidelines for designing and conducting PCESs [29]. This matrix contains 14 items for research practices grouped under 3 types of validity threats that the practices are designed to address: (1) threats to internal validity, (2) threats to construct validity, and (3) threats to external validity (S2 Table).

For items like randomization or sample size, practices pertain to individual studies and can be scored relatively easily at the PCES level (Table 2). Other items, such as whether mechanistic or replication studies were performed, pertain less to individual studies than to a package of evidence and were thus rated at the IB level (Table 2). The 14 items and their clarifying questions were to be rated as reported, not reported, or not applicable. The scoring criteria are presented in more detail in S2 Table.

To score whether each PCES demonstrated an effect or not, we extracted inferential statistics (effect sizes and significance values) or narrative wording for results. When inferential tests were performed, we defined “demonstration of effect” based on whether the 95% confidence interval excluded the null or whether *p*-values were reported as being less than or equal to 0.05.

All rating and scoring of PCESs and IBs was piloted independently by 3 authors (WWLC, SW, and CF) in an initial sample of 10 IBs; these IBs contained 77 PCESs in total. Unclear ratings and initial disagreements were discussed with JK and DS, and the scoring criteria were slightly modified. Once the final scoring sheet was agreed upon, SW and WWLC selected and scored PCESs independently from a second random sample of 10 IBs including 117 PCESs. For this independent rating, we found a discordance between 0% and 16% per item, resulting in a mean inter-rater reliability of 94% (S3 Table). Thereafter, WWLC selected and rated the remaining 59 IBs and SW 12 IBs. The 18 IBs that lacked any PCESs were not further analyzed. All unclear cases from this third round of analysis were discussed with all other authors and resolved.

Some IBs cite peer-reviewed publications including further information on the conduct and results of their PCESs. We identified these publications (*n* = 80) and applied the same matrix of 14 items to extract practices addressing validity threats from full-text publications (SW rated 56 publications, and SS rated 24 publications). Again, all unclear ratings were discussed with all other authors and could be resolved.

Descriptive statistics were applied.

## Supporting information

S1 Table Text examples for preclinical efficacy studies demonstrating no effect. (DOCX)

S2 Table Raw data and scoring criteria. (XLSM)

S3 Table Results for inter-rater reliability. (XLSM)

## Abbreviations

ARRIVE: Animal Research: Reporting of In Vivo Experiments
BfArM: Bundesinstitut fuer Arzneimittel und Medizinprodukte
BMJ: British Medical Journal
EMA: European Medicines Agency
FDA: Food and Drug Administration
IB: investigator brochure
ICH: International Council for Harmonisation of Technical Requirements for Pharmaceuticals for Human Use
IRB: institutional review board
PCES: preclinical efficacy study
