# Supplementary material for: Preclinical efficacy studies in investigator brochures: Do they enable risk–benefit assessment?
Source: PLoS Biol. 2018 Apr 5;16(4):e2004879. doi: 10.1371/journal.pbio.2004879 (PMC5886385; doi:10.1371/journal.pbio.2004879)
Supplement: S1 Table — (DOCX) [file pbio.2004879.s001.docx]

Table S1: Text examples for studies with negative results

| IB7 | Due to good tolerability but lack of efficacy, the dose was increased to 75 mg/kg during the study at the beginning of the 3rd treatment cycle. Moderate activity was observed with single-agent [substance x], which could be improved by the addition of [substance y] at half of its MTD […]. However, the combination was not superior to single-agent [substance x] in this model. The combination as well as both single-agent treatment regimens were well tolerated. |
| --- | --- |
| IB12 | Lack of Efficacy of [substance x] in […] Model […] Either as Single Agent or Added to [substance y] (Study title) |
| IB21 | 0% efficiacy (results table) |
| IB22 | [Substance x] had no effect on the growth of […] tumors in the transgenic […] mouse model |
| IB49 | Conversely, the following models harboring […] mutations […], a […] mutation […], […] mutations […], […] mutations […], or […] mutations […] did not respond to [substance x] treatment. |
| IB54 | Protection was no longer observed when [substance x] administration started on Day 8 or 12 after s.c. inoculation of tumor/PBMC mixtures. Loss of activity with delay of administration may relate to the short half life of […] in […] mice. |
| IB77 | In contrast, early in vivo combination studies using […] with [substance x]  being given 15-20 min before or 24 h after the cytotoxic showed no combinational efficacy. |
| IB79 | Oral administration of [substance x] at 30 mg/kg/day for 14 days did not increase […], as compared to the vehicle group. |
| IB87 | Although [substance x] did not affect the number of polyps that formed in the gut of […] mice a significant increase (approximately 40%) in the diameter of the polyps was noted in [substance x]–treated animals |
| IB91 | In the […] cancer (which did not respond to [substance x] in vitro) xenograft model, no significant inhibition was seen with monotherapy or combination treatment. |
